# Pore-Scale Modeling of Microporous Layer for Proton Exchange Membrane Fuel Cell: Effective Transport Properties

**DOI:** 10.3390/membranes13020219

**Published:** 2023-02-10

**Authors:** Heng Zhang, Xuanyu Shao, Zhigang Zhan, Mrittunjoy Sarker, Pang-Chieh Sui, Po-Ya Abel Chuang, Mu Pan

**Affiliations:** 1 Foshan Xianhu Laboratory of the Advanced Energy Science and Technology Guangdong Laboratory, Xianhu Hydrogen Valley, Foshan 528200, China; 2 State Key Laboratory of Advanced Technology for Materials Synthesis and Processing, Wuhan University of Technology, Wuhan 430070, China; 3 Mechanical Engineering, University of California, Merced, CA 95343, USA; 4 Institute for Integrated Energy Systems, Department of Mechanical Engineering, University of Victoria, Victoria, BC V8W 2Y2, Canada

**Keywords:** microporous layer (MPL), stochastic numerical method, transport properties, pore scale model, Lattice Boltzmann method, compression strain

## Abstract

**Highlights:**

**What are the main findings?**

- Stochastic numerical method is carried out to reconstruct the realistic microstructure of microporous layer (MPL) in a PEMFC.
- Computation of effective transport properties by Pore Scale Model and lattice Boltzmann Method is carried out.

**What is the implication of the main finding?**

- The relationships between effective transport properties and compression strain are summarized.
- Correlations between effective transport properties and compression strain are obtained and used to predict PEMFC performance.

**Abstract:**

A microporous layer (MPL) is a transition layer with a porous material structure, located between the gas diffusion layer (GDL) and catalyst layer (CL) in a proton exchange membrane fuel cell (PEMFC). It not only significantly improves electron transfer and heat conduction in membrane electrode assembly, but also effectively manages liquid water transport to enhance the fuel cell performance. The MPL is usually coated on one side of the GDL. The fragile nature of MPL makes it challenging to characterize the effective transport properties using experimental methods. In this study, a stochastic numerical method is implemented to reconstruct the three-dimensional microstructure of an MPL consisting of carbon particles and PTFE. The reliability of the MPL reconstructed model is validated using experimental data. The relationship between the effective transport properties and the compression strain is obtained using the Pore Scale Model (PSM), while the relationship between the liquid water saturation and capillary pressure is solved by Lattice Boltzmann Method (LBM). The effective transport properties in the MPL are then imported into the two-phase flow fuel cell model. It is found that the effective transport parameters in MPL obtained by PSM and LBM can improve the accuracy of the model calculation. This study provides an effective method to reconstruct the microstructure of MPL that can generate precise MPL transport parameters for utilization in various PEMFC performance prediction models.

## 1. Introduction

With the increasing shortage of fossil fuels and environmental factors associated with burning them, it is really important to develop a new and alternative energy device that offers environmental protection by offering low or zero emission [1]. In recent decades, proton exchange membrane fuel cell (PEMFC) has gained great attention as a new type of energy device. It can be regarded as one of the most promising energy conversion devices due to its fast start-up, high power density, high energy conversion efficiency, it is environment-friendly, and pollution-free features [2,3].

A PEMFC comprises a membrane electrode assembly (MEA), a gas diffusion layer (GDL), and bipolar plates (BPP). The MEA is the key part of a PEMFC, which traditionally consists of catalyst layers (CL) and a proton exchange membrane (PEM) [4,5]. To effectively enhance the water-thermal management capability in the PEMFC, GDL is often incorporated with an additional layer known as the microporous layer (MPL), which is located between GDL and CL [2]. MPL is made of a carbon black, hydrophobic agent e.g., Polytetrafluoroethylene (PTFE), solvent, and pore-forming agent [6]. MPL provides a path for mass (e.g., gas-liquid) transport, heat transfer, and electron conduction. In addition, it can effectively reduce the contact electrical and thermal resistance between GDL and CL due to its compliant and compact structure.

The incorporation of the MPL can increase the capillary pressure due to its relatively low porosity, small pore size, and large average contact angle. In addition, the PTFE particles in the MPL can provide sufficient hydrophobicity so that the pores remain open as stable gas and liquid water paths. In sum, MPL not only ensures consistent reactant flow to the CL for electrochemical reactions to happen but also effectively prevents CL from being flooded by liquid water. Moreover, MPL provides mechanical support to the CL and the membrane, prevents catalyst particle penetration into the GDL, and protects the membrane from damage by the GDL carbon fibers [7]. The MEA is compressed and deformed during the fuel cell assembly [8], and the extent of deformation of the MPL is less than that of the GDL due to MPL’s greater Young’s modulus and lower thickness. Nevertheless, the change in physical properties of the MPL due to compression may lead to variation in transport properties, and this variation has a non-negligible effect on the mass and heat transfer during fuel cell operation [9]. As previously studied by Zhang et al. [10], at an operating current density of 1.14 A/cm^2^, the oxygen molar concentration changes by 2.2 mol/m^3^, and the temperature changes by 0.3℃ in the GDL, while the oxygen molar concentration changes by 3.8 mol/m^3^ and the temperature changes by around 1℃ in the MPL. The higher the current density, the more pronounced the effect of MPL on the distribution of reactant oxygen, liquid water, and temperature. Therefore, developing an in-depth and comprehensive understanding of the MPL microstructure and transport properties is crucial to help with enhancing PEMFC performance and durability.

A handful amount of experimental and numerical studies have been conducted to study the transport properties in MPL. X-ray tomographic microscopy has been used by Chen et al. to experimentally characterize the MPL for obtaining their morphological parameters [11]. A newly advanced experimental method has been proposed to investigate the structural evolution and design optimization of the MPL [6]. Li et al. [12] experimentally studied the effect of six different types of carbon black as cathode MPL and the use of polyvinylidene fluoride hexafluoropropylene copolymer P(VDF-HFP) instead of the traditional PTFE as the hydrophobic agent on the cell performance. Simon et al. [13] experimentally investigated the impact of MPL pore sizes on liquid water transport. They concluded that the liquid water in MPL tends to be transported through the larger pores, whereas the smaller pores are used for transporting the oxygen molecules. Tabe et al. [14], Prass et al. [15], Nagai et al. [16], Lee et al. [17], and Spernjak et al. [18] experimentally studied the transport of oxygen and liquid water in the MPL and its effect on cell performance, as well as adjusting the content and distribution of carbon particles and hydrophobic agents to optimize the PEMFC performance. In addition to experimental methods, the simulation tool is also used by many researchers for understanding the MPL transport properties. Xu et al. [19] developed a 1-D, non-isothermal, two-phase model to investigate the liquid water transport in cathode GDL and MPL. Lattice Boltzmann Method (LBM) was applied to analyze the effects of mechanical stress and wettability heterogeneity of GDL coated with MPL on liquid water transport by Ira et al. [20]. Hou et al. [21] created a 3D LBM model to study the effect of MPL on liquid water transport by taking hydrophobicity, pore size distribution, and MPL structure into consideration. They found that smaller pore size and the presence of more hydrophobic material induce greater capillary pressure. Zhou et al. [9] implemented a multi-dimensional, non-isothermal, two-phase model to understand the function of MPL in improving the performance of PEMFC. Hannach et al. [22] developed a 3D stochastic reconstructed microstructure of MPL to compute the effective transport properties and thermal conductivity. Tadbir et al. [23], Zhang et al. [24], and Sadeghifar et al. [25] numerically investigated the role of MPL in PEMFC and analyzed the mechanism of transport in MPL.

Although there are many studies on the transport properties of MPL in recent years, the effect of mechanical stress on the MPL transport properties has not been investigated experimentally likely due to the mechanical instability of the MPL [22]. The numerical simulation could be a reliable method for analyzing the effect of mechanical stress on the MPL transport properties. MPL reconstruction to obtain a realistic microstructure is the basis for performing such studies. Becker et al. [26] and Zamel et al. [27] reconstructed the microstructure of MPL by stochastic numerical method. However, PTFE was neglected and not fully reconstructed in their work. Hannach et al. [22] obtained a realistic 3D MPL microstructure, Lee et al. [17], Deng et al. [28], Bock et al. [8], Aoyama et al. [29], and Nozaki et al. [30] studied the transport of gas-water-heat-electricity in MPL. However, only a few of the published works considered the effect of mechanical compression on the MPL transport properties.

To address the knowledge gap, a stochastic method is used to reconstruct the MPL microstructure, and the reconstruction results are validated by the distribution of porosity and pore size obtained experimentally. The relationship between the effective gas diffusivity, thermal and electrical conductivity, and compression strain in the in-plane and through-plane directions are obtained by the pore scale model (PSM). In addition, the LBM model is applied for solving the relationship between effective liquid water permeability and compression stain as well as the liquid water saturation as a function of capillary pressure for both in-plane and through-plane directions. All of these relations are fitted into mathematical expressions that can provide accurate input parameters for PEMFC simulation analysis. The results of this study can provide both a robust approach to reconstructing the MPL microstructure and insights into an accurate prediction of transport properties in the MPL.

## 2. Numerical Analysis Procedure

### 2.1. 3D Reconstruction of the MPL

**Figure 1:**
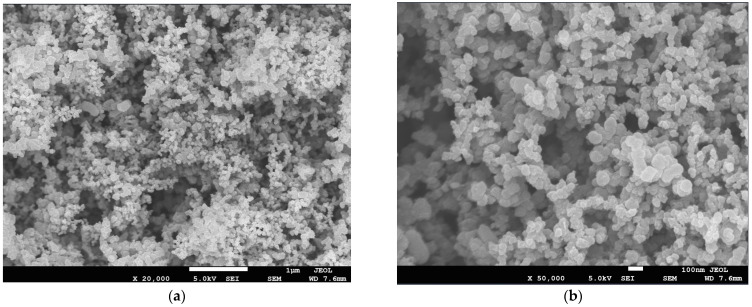
SEM images of the MPL at the resolutions of (**a**) 1 μm and (**b**) 100 nm.

To obtain a realistic MPL microstructure, a field emission scanning electron microscope (SEM) and X-MAX N80 energy spectrometer JSM-7500F are used. SEM images of MPL microstructure with resolutions of 1 μm and 100 nm and magnifications of 20,000 and 50,000 are utilized for evaluating the morphology of the composition and the distribution of pores as shown in Figure 1. The analyzed microstructure shows a porous structure and consists of carbon particles and PTFE agglomerates, which are spherical-like.

**Figure 2:**
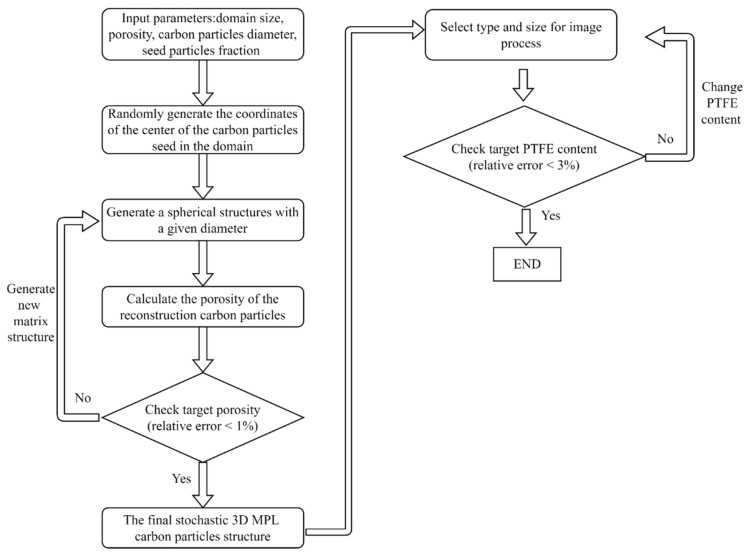
The flowchart of stochastic numerical reconstruction of 3D MPL microstructure.

For the stochastic numerical reconstruction method, the carbon particles are simulated using a tailor-made reconstruction code. The input parameters of the reconstruction code include domain size, porosity, carbon particle diameter, seed particle fractions, and the degree of overlap in contact. The detailed numerical reconstruction processes in this study are shown as a flowchart in Figure 2. First, a number of seed particles separated from each other without contacting and overlapping are generated and randomly distributed in the predefined computational domain. Then, the remaining carbon particles are added to the computational domain with these seed particles as the center and starting point of randomly generated carbon particle agglomerates. It needs to be ensured that there is a certain part of physical contact between these newly generated carbon particles and the preexisting carbon particles. The number and placement of the carbon particles are iterated in the reconstruction code until the final porosity reaches the preset target porosity. To ensure the feasibility of MPL reconstruction, it is assumed that all carbon particles are spheres with the same diameter, and the degree of overlap between carbon spheres also remains the same. Subsequently, PTFE can be added to the carbon particles using the morphological software AVIZO for generating the final MPL microstructure [22,31].

In this study, an MPL microstructure is iteratively generated with a computational domain of 200 × 200 × 200 pixels (1 pixel = 10 nm), which is initially filled with empty space, carbon particles, and PTFE. The final MPL structure has a porosity of 51.8% with a weight fraction of PTFE of 40 wt.% [6]. In the reconstruction process, there are a few morphological parameters that need to be determined to obtain a distribution of carbon particles close to the actual MPL materials. Deriving from realistic MPL experimental data, these parameters include the fraction of seeded particles to be 0.1, and the degree of overlap between particles to be 0.25 [22,31].

### 2.2. Governing Equations of PSM

The pore phase in the MPL provides a transport channel for gas species, whereas electrons and heat are conducted through the entire solid phase consisting of carbon and PTFE particles, and the thermal conduction occurs in the entire computational domain including the solid and pore phases. The governing equations for solving the effective transport of gas-electricity-heat in the MPL are employed via the PSM. There are three assumptions in this model: (1) the convection effect is ignored, meaning binary diffusion is the only mechanism for gas transport, (2) air pressure is set to be 200 kPa for the computational domain, and (3) no condensation as liquid water transport will be considered in the LBM simulation later [32,33].

Knudsen diffusion is considered in the MPL since the average pore size may be smaller than the mean-free path of gas particles. The effective gas transport parameters are solved using the following equations [34,35]:

(1) $$\begin{array}{c} \nabla x_{O_{2}}=\frac{RT}{p}\left(\frac{x_{O_{2}}j_{H_{2}O}-x_{H_{2}O}j_{O_{2}}}{D_{O_{2}-H_{2}O}}+\frac{x_{O_{2}}j_{N_{2}}-x_{N_{2}}j_{O_{2}}}{D_{O_{2}-N_{2}}}-\frac{j_{O_{2}}}{D_{O_{2},Kn}}\right) \end{array};\;\begin{array}{c} \nabla j_{O_{2}}=0\; \end{array}$$

(2) $$\begin{array}{c} \nabla x_{H_{2}O}=\frac{RT}{p}\left(\frac{x_{H_{2}O}j_{O_{2}}-x_{O_{2}}j_{H_{2}O}}{D_{O_{2}-H_{2}O}}+\frac{x_{H_{2}O}j_{N_{2}}-x_{N_{2}}j_{H_{2}O}}{D_{H_{2}O-N_{2}}}-\frac{j_{H_{2}O}}{D_{H_{2}O,Kn}}\right) \end{array};\;\begin{array}{c} \nabla j_{H_{2}O}=0\; \end{array}$$

(3) $$\begin{array}{c} \nabla x_{N_{2}}=\frac{RT}{p}\left(\frac{x_{N_{2}}j_{O_{2}}-x_{O_{2}}j_{N_{2}}}{D_{O_{2}-N_{2}}}+\frac{x_{N_{2}}j_{H_{2}O}-x_{H_{2}O}j_{N_{2}}}{D_{H_{2}O-N_{2}}}-\frac{j_{N_{2}}}{D_{N_{2},Kn}}\right) \end{array};\;\begin{array}{c} \nabla j_{N_{2}}=0\; \end{array}$$

where $x_{i}$ is the mole fraction of species i, $j_{i}$ is the flux of species i, $D_{i-j}$ is the binary diffusivity, and $D_{i,Kn}$ is the Knudsen diffusivity of species i. The binary diffusivities are functions of temperature and pressure and are summarized in our previous study [33]. The Knudsen diffusivity is computed by Equations (4)–(6) [36]:

(4) $$D_{O_{2},\;Kn}=4850d_{p}\sqrt{\frac{T}{M_{O_{2}}}}$$

(5) $$D_{N_{2},\;Kn}=4850d_{p}\sqrt{\frac{T}{M_{N_{2}}}}$$

(6) $$D_{H_{2}O,\;Kn}=4850d_{p}\sqrt{\frac{T}{M_{H_{2}O}}}$$

where $d_{p}$ represents the pore diameter of MPL, $M_{O_{2}}$, $M_{N_{2}}$, and $M_{H_{2}O}$ is the molecular mass of oxygen, nitrogen, and water vapor, respectively.

Obtaining the mean pore size in the MPL is key to solving Knudsen diffusivity. Some studies used the average pore diameter size to solve the Knudsen diffusivity in the whole domain, and some determined the pore size based on the distribution of porosity and pore diameter. However, none of these methods accurately calculated the Knudsen diffusivity since the smaller sized pores have a more pronounced Knudsen effect as compared to the larger pores. The PSM, as well as the LBM introduced in the next section, are based on an extension in three dimensions, x-y-z, which is used to study the Knudsen diffusion in the MPL. It allows the calculation of the pore size in each pore cell. A detailed description can be found in the literature [34,35].

The electron and heat flux in the MPL are computed by ohm’s law and Fourier’s law as presented in Equations (7) and (8), respectively. The source term of electron flux is 0, and the ohmic heat generated due to the conduction of charged particles in the solid phase is considered.

(7) $$\begin{array}{c} j_{e}=-\sigma_{e}\nabla \phi_{e} \end{array};\;\begin{array}{c} \nabla j_{e}=0 \end{array}$$

(8) $$\begin{array}{c} j_{T}=-\lambda \nabla T \end{array};\;\nabla j_{T}=\frac{{\left(\nabla \phi_{e}\right)}^{2}}{\sigma_{e}}$$

where *j* is the flux, subscripts *e*, and *T* denotes electrons and heat, respectively; σ is the conductivity, ϕ is potential, λ is thermal conductivity, $\lambda_{solid}$ denotes solid phases thermal conductivity, and $\lambda_{air}$ denotes air thermal conductivity. The $\lambda_{air}$ is computed by Equation (9):

(9) $$\lambda_{air}\left[W\;m^{-1}\;K^{-1}\right]=\left(-0.099489\alpha +2.0\right)\left(0.022423\left(T-273.15\right)+13.27\right)\times 10^{-3}$$

Relative humidity, α, is defined as the ratio of water vapor pressure to saturation pressure, and calculated using the following expression:

(10) $$\alpha =\frac{c_{H_{2}O}RT}{p_{sat}}$$

where $c_{H_{2}O}$ is the molar concentration of water vapor, R is the universal gas constant, *T* is the temperature, $p_{sat}$ is the saturation pressure. $p_{sat}$ is computed by the following formula:

(11) $$p_{sat}\left[Pa\right]=a_{1}+a_{2}\left(T-273.15\right)+a_{3}{\left(T-273.15\right)}^{2}+a_{4}{\left(T-273.15\right)}^{3}$$

where the coefficients $a_{1}$, $a_{2},\;a_{3}$, and $a_{4}$ are constants obtained by curve fitting: $a_{1}=-2846.4,\;a_{2}=411.24,\;a_{3}=-10.554$, and $a_{4}$ = 0.16636.

The thermal conductivities of the solid phase, including carbon particles and PTFE, are set to be a constant in the PSM. For carbon particles: the thermal conductivity is 100 W m^−1^ K^−1^, and the electrical conductivity is 15,000 S m^−1^; for PTFE: the thermal conductivity is 0.2 W m^−1^ K^−1^, and the electrical conductivity is close to zero [8,17,33].

In the final 3D MPL obtained by numerical reconstruction, the two opposite faces in the in-plane direction are set as Dirichlet boundary conditions when solving for the in-plane transport properties, and the other four sides are set to be periodic boundary conditions. Similarly, the Dirichlet boundary conditions are applied to the two opposite faces in the through-plane direction when the through-plane transport properties are solved, and the other four faces are also set to be as periodic boundary conditions. Heat conduction, electron transport, and gas diffusion are driven by the difference in temperature, potential, and molar concentration in the PSM. Therefore, the difference between the two opposite faces of temperature, potential, and gas concentration are applied to be 0.1 K, 0.01 V, and 0.1 mol m^−3^ to obtain effective thermal conductivity, electrical conductivity, and gas diffusivity, respectively. The values of the boundary conditions on these two sides are listed in Table 1 [4].

The effective thermal conductivity, electrical conductivity, and gas diffusivity can be obtained by the following equation:

(12) $$\begin{array}{c} V_{eff}=-\frac{F_{g}\cdot L}{\left(b_{2}-b_{1}\right)} \end{array}$$

where $V_{eff}$ represents the effective transport parameter including thermal conductivity, electrical conductivity, and gas diffusivity, $F_{g}$ is the total flux of heat, electron, and gas of the computational domain which can be obtained by PSM simulation program code, L is the domain length, and $b_{1}$ and $b_{2}$ represent the prescribed boundary conditions of two opposite sides as shown in Table 1.

### 2.3. Governing Equations of LBM

The transport of liquid water in the entire MPL reconstructed computational domain is solved by LBM [37]. The contact angle of the carbon particles and PTFE are set to be 80° and 130°, respectively [38]. A pressure gradient is applied on two opposing faces in the computational domain to drive the transport of liquid water in the MPL. Liquid water permeates dynamically into the MPL until the LBM simulation converges and the liquid water transport reaches a stable equilibrium state.

The Navier–Stokes and the Cahn–Hilliard equations are used to compute the transport of multi-phase and are expressed in the following equations [39]:

(13) $$\frac{\partial \rho u}{\partial t}+\nabla \cdot \rho uu=-\nabla \cdot p+\eta \nabla^{2}u+F$$

(14) $$\frac{\partial \varphi }{\partial t}+\nabla \cdot \varphi u=M\nabla^{2}\mu$$

In the LBM simulation program code used in this study, a specified amount of liquid water is placed on the higher-pressure boundary side of the MPL reconstructed domain, and the pressure of the opposite side is lower. Therefore, there is a pressure gradient that can drive the transport of liquid water. The periodic velocity field is the boundary condition for the other four sides of the domain. More detailed descriptions of LBM simulations can be found in the literature [33,37,40].

## 3. Results and Discussions

### 3.1. Reconstructed MPL Microstructure

**Figure 3:**
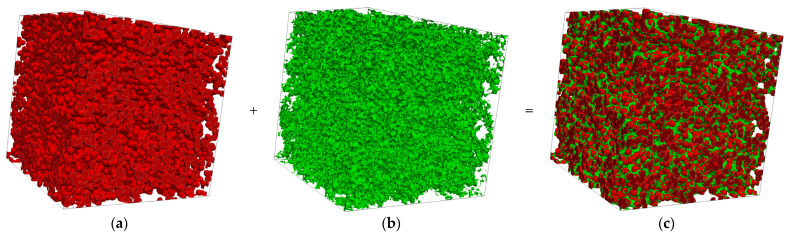
3D stochastic reconstruction rendering of MPL microstructure: (**a**) carbon particles, (**b**) PTFE, (**c**) final MPL.

The process of the 3D microstructure reconstruction of MPL is shown as a flowchart in Figure 2. The first step of reconstructing MPL is to reconstruct the carbon particles followed by adding PTFE to the carbon particles. As shown in Figure 3a, the carbon particles are reconstructed by a customized numerical program code. Subsequently, 40 wt.% of spherical-like PTFE [6,18] is added to the carbon particles using AVIZO, as presented in Figure 3b. The final 3D MPL structure containing carbon particles and PTFE is obtained by the numerical stochastic method. The computational domain size of the MPL reconstruction microstructure is 2 × 2 × 2 µm^3^ and is shown in Figure 3c.

**Figure 4:**
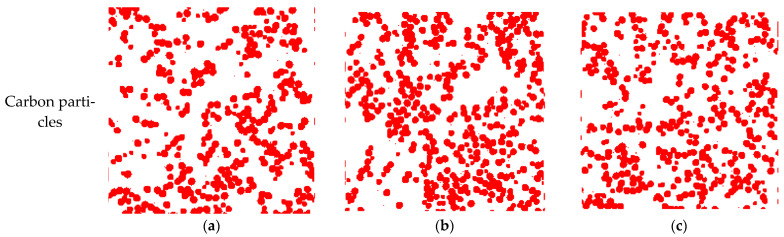

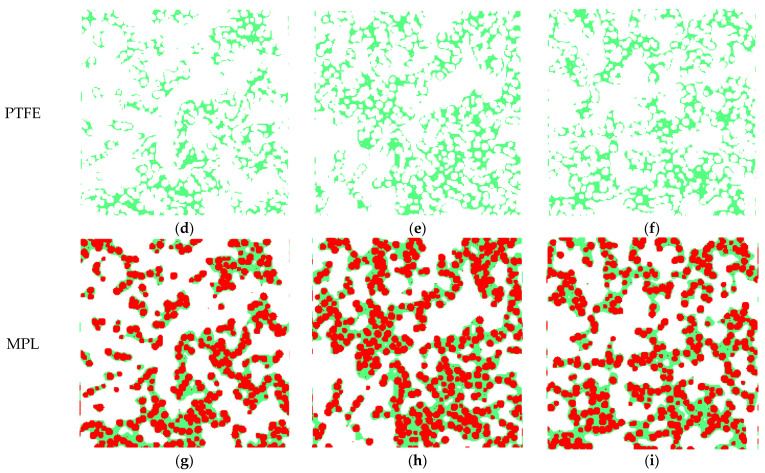
Distribution of (**a**–**c**) carbon particles, (**d**–**f**) PTFE, and (**g**–**i**) MPL on three different sections of (**a**,**d**,**g**) slice 25, (**b**,**e**,**h**) slice 100, and (**c**,**f**,**i**) slice 175.

To better illustrate the distribution of the solid phase in the MPL, three different in-plane (x-y) sections are selected for comparison. It can be observed from Figure 4 that the shape of carbon particles is spherical-like, the PTFE is located at the junction of the carbon particles and adheres to the existence on the surface of carbon particles, which is consistent with the reference [22]. These reconstructed distributions show good agreement with those in the SEM images of the MPL, which validates the credibility of the reconstructed MPL morphology.

**Figure 5:**
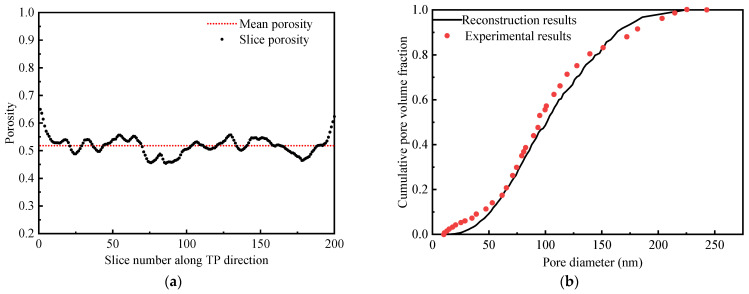
(**a**) The distribution of mean porosity and slice porosity along the *z*-axis direction; (**b**) Comparison of pore size distributions from simulation studies and experimental data.

After the stochastic reconstruction, the final distribution of average porosity (51.8%) and the local in-plane slice porosity along the through-plane direction are shown in Figure 5a. The porosity of each slice is the ratio of the pore phase to the overall volume; it is inversely related to the presence of solid phases including carbon particles and PTFE on each slice. It can be found that the local porosity in the through-plane direction ranges from 45% to 65%. The porosity tends to be higher on both sides of the computational domain. This is because the near-surface is an open space for the computational domain. Moreover, PTFE will be more distributed in the area with more carbon particles, which can further lead to less porosity in the central area of the computational domain.

In addition to the porosity distribution, another index to measure the reliability of MPL reconstruction is the pore size distribution, which can be obtained by simulated reconstruction and validated by experimental characterization as represented in Figure 5b. Comparing the simulated data with experimental characterization shows a good agreement with the realistic MPL pore size distribution [22]. The mean pore size of reconstructed MPL is around 100 nm and ranges from 10 nm to 250 nm. The results of the distribution of porosity and pore distribution in the MPL obtained by numerical simulations in this study show good agreement with the results in the literature [21,22], proving the correctness and reliability of the reconstructed MPL.

### 3.2. Effective Gas Diffusivity, Thermal and Electrical Conductivity

After we obtain the final 3D MPL microstructure by stochastic numerical reconstruction method, PSM is then used to solve the effective gas diffusivity, thermal conductivity, and electrical conductivity. The assembly forces are generated during the assembly of the fuel cell stack, and it not only causes deformation in GDL, which has a low Young’s modulus but also generates mechanical deformation in the MPL [8]. The deformation can cause changes in the porosity of the MPL, ultimately affecting the transport properties including tortuosity, effective gas diffusivity, and thermal and electrical conductivities in the MPL.

**Figure 6:**
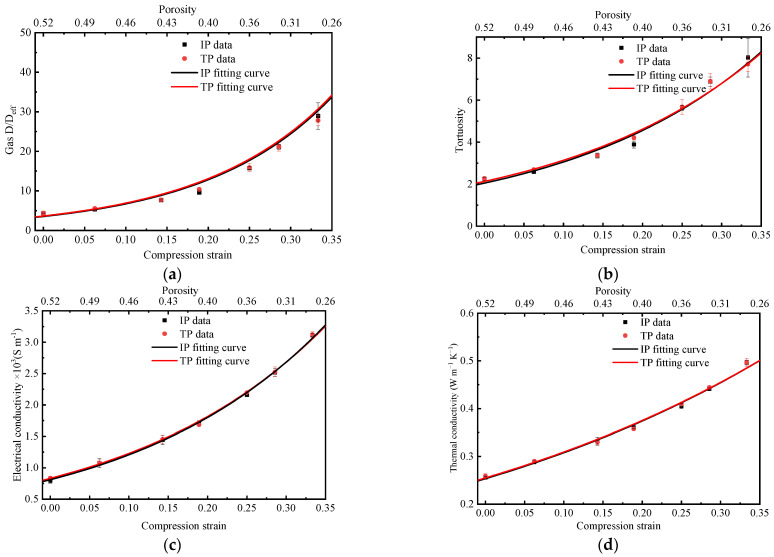
The relationship between in-plane and through-plane (**a**) effective gas diffusivity, (**b**) tortuosity, (**c**) electrical conductivity, and (**d**) thermal conductivity as functions of compression stain or porosity in the MPL.

The effect of compression strain, ranging from 0 to 0.35, on the D/D_eff_ and tortuosity in MPL are investigated, with results represented in Figure 6a,b, respectively. The mechanical compression effect in the MPL can be represented by the change of porosity in this study and the relationship between the porosity after compression [33]. The compression strain can be solved using the following equation [41]:

(15) $$\varepsilon_{comp}=\frac{\varepsilon_{0}-\varsigma }{1-\varsigma }$$

where ς represents compression strain, $\varepsilon_{0}$ and $\varepsilon_{comp}$ represent the porosity without compression and with compression, respectively.

The tortuosity τ in the MPL can be obtained using the following equation:

(16) $$\frac{D}{D_{eff}}=\frac{\tau }{\varepsilon }$$

where *D* and $D_{eff}$ is nominal and effective diffusivity, respectively.

According to the post-processing of Equation (16), the relationship between tortuosity and compression strain in MPL can be found and is shown in Figure 6b. Since both carbon particles and PTFE are spherical-like, MPL exhibits obvious isotropic behavior. As observed, the effective gas diffusivity decreases with the increase in compression strain, while tortuosity increases with increasing strain. This is because as the compression strain increases, the porosity of MPL decreases, and the gas transport resistance increases. When the compression strain is increased from 0 to 0.35, the tortuosity in the MPL increases from 2 to 8. The relationship between *D*/*D_eff_*, tortuosity, and compression strain or porosity are fitted into correlations as summarized in Table 2.

Electrical and thermal conductivities of MPL are the measure of its ability to conduct electrons and heat. It can significantly affect the distribution of current density and temperature, which can ultimately influence the fuel cell performance. The effective electrical conductivity and thermal conductivity as a function of compression strain in the in-plane and through-plane directions in MPL are solved by PSM simulation and results are shown in Figure 6c,d, respectively. As can be seen, when the compression rate changes from 0 to 0.35, the electrical conductivity in MPL increases from 750 S m^−1^ to 3250 S m^−1^, and the thermal conductivity increases from 0.25 W m^−1^ K^−1^ to 0.5 W m^−1^ K^−1^. The relationship between thermal and electrical conductivities as functions of compression strain or porosity are also fitted and are summarized in Table 2.

### 3.3. Effective Liquid Water Permeability and Saturation

**Figure 7:**
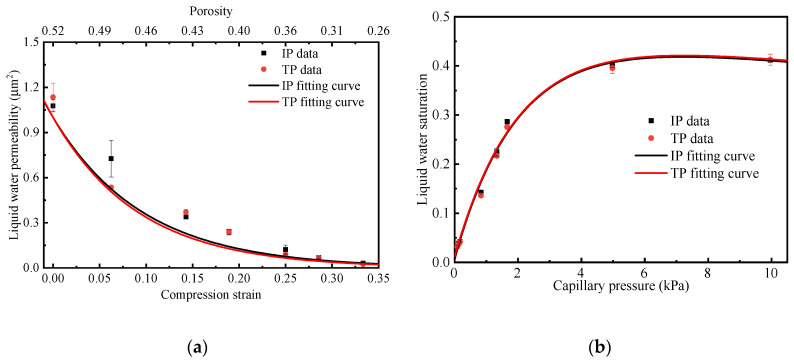
(**a**) The relationship between permeability and compression strain or porosity of the MPL, and (**b**) the relationship between liquid water saturation and capillary pressure in the in-plane and through-plane direction of MPL.

Since MPL plays a crucial role for water transport in a PEMFC, it is essential to gain a deeper understanding of two-phase flow distribution in the MPL. In this study, LBM is used to simulate the relationship between in-plane and through-plane liquid water permeability and compression strain, the outcomes of which are shown in Figure 7a. The Kozeny-Carman equation is used to solve the permeability in the porous media [42], which accounts for the permeability that is positively related to porosity and pore size, and negatively related to tortuosity. From Figure 7a, it can be found that the liquid water permeability decreases significantly with the increase of compression strain due to the decrease in porosity and pore size. The permeability of liquid water is 1.1 μm^2^ when the MPL is not compressed, and permeability decreases to 0.05 μm^2^ under a compression strain of 0.35. It indicates that mechanical pressure increases the transport resistance of liquid water in the MPL, making the porous media under the ribs more likely to accumulate water as compared to the media under the channel [20,43].

Liquid water saturation in the MPL can also be computed by the LBM. The capillary pressure increases with the increasing contact angle of the solid phase and is inversely proportional to the hydrodynamic radius in the porous media [44]. The relationship between liquid water saturation and capillary pressure is shown in Figure 7b. The trend of liquid water saturation with capillary pressure is similar along the in-plane and through-plane directions, which corresponds to the isotropic properties of the MPL. When the capillary pressure is less than 5 kPa, the liquid water saturation increases almost linearly to about 0.4 with increasing capillary pressure. However, when the capillary pressure is greater than 5 kPa, the liquid water saturation remains almost stable at about 0.4. This indicates that the liquid water in the MPL has reached a dynamic equilibrium due to most of the hydrophilic carbon interface being fully saturated. This finding shows how the MPL effectively prevents MEA from being flooded in a PEMFC [7,45]. The relationship between saturation and capillary pressure in the in-plane and through-plane directions are fitted and are summarized in Table 2.

### 3.4. Application of Effective Transport Parameters in an MPL

In our previous study, a two-dimensional, non-isothermal two-phase flow PEMFC model was developed, considering mechanical stress, electrochemical reactions, heat and mass transfer, and liquid water transport [10]. Empirical formulas for the effective transport parameters in porous media were used, which resulted in insufficient accuracy when compared with experimental data. To obtain more accurate input parameters of transport properties, a detailed investigation is required to be performed to solve the relationship between transport properties and compression strain and/or porosity. For GDL, the PSM and LBM were used to solve for the effective transport parameters in the in-plane and through-plane directions, as accomplished in our previous study [33], but with the implementation of improved correction for the transport properties parameters as taken from the mechanical stress deformation and liquid water saturation in the GDL. Similar to GDL, this current study is able to obtain the relationship between transport properties and porosity caused by mechanical compression in the MPL.

**Figure 8:**
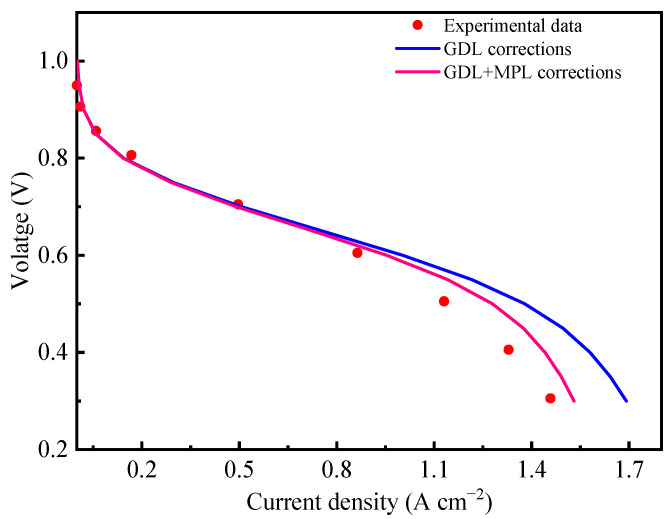
Comparison of experimental and simulation polarization curves after correction of the effective transport properties.

To validate the reliability of these input parameters for GDL and MPL for the computational model, the input transport parameters for GDL and MPL are imported to the two-phase model. In this model, the GDL corrects for transport parameters due to deformation and liquid water saturation, and the MPL corrects for transport parameters due to the change in porosity caused by the formation of the liquid water saturation. The simulation results are compared with fuel cell experimental data as shown in Figure 8. The experiment was carried out on an automated HEPHAS PEMFC test station with a cell-activated area of 10 cm^2^ and using a straight-parallel channel design for the flow field. The catalyst-coated membrane (CCM) consisting of polymer electrolyte Nafion NR211 membrane and electrode was purchased from WUT Energy Co., Ltd. The CL has a platinum loading of 0.1 mg cm ^−2^. The GDM consists of Toray TGP-H-060 carbon fiber paper coated with MPL with a PTFE loading of 40 wt.%. Hydrogen and air were fed at the anode and cathode, respectively, with the same stoichiometric ratio of 2 on the anode and cathode. The cell operating conditions were 80 °C, 200 kPa abs., and 60% RH. The cell was operated with a potentiostatic control from OCV down to 0.30 V. As can be observed from the figure, the accuracy of the computational model is significantly improved at the high current density region after the effective transport parameter correction for the change in porosity due to saturation in the MPL by using the equations in Table 2.

The improvement at a high current density of incorporating both GDL and MPL transport properties is mainly due to increasing liquid water saturation in both GDL and MPL, which requires accurate MPL transport properties. Therefore, the effective transport property corrections in MPL obtained in this study can effectively improve the accuracy of model prediction, especially at high current density operations. In our future work, we plan to consider the mechanical deformation of MPL in the model, as well as the parameter correction in CL and PEM, to provide systematic parameter input for theoretical simulation studies in PEMFC.

## 4. Conclusions

In this study, a stochastic numerical method is implemented to reconstruct the 3D microstructure of MPL. PSM is used to solve the relationship between effective transport properties including gas diffusivity, tortuosity, electrical and thermal conductivities, and compression strain. Additionally, LBM is applied to obtain the relationship between liquid water permeability and compression strain, and between liquid water saturation and capillary pressure. In addition, the relationships between the effective transport properties and compression strain are fitted and summarized as functional correlations. MPL exhibits isotropic behavior in its transport properties due to spherical-like constituents. The tortuosity, electrical and thermal conductivities of the MPL increase with increasing compression strain. However, the liquid water permeability decreases with increasing compression strain. The saturation in MPL increases with the increase of capillary pressure, and the transport of liquid water reaches a dynamic steady state with a saturation of about 0.40 when the capillary pressure is increased to around 5 kPa due to most of the hydrophilic carbon interface is fully saturated. The corrected formulas of the effective transport properties parameters in MPL obtained by PSM and LBM can effectively improve the accuracy of the two-phase PEMFC model, especially in the concentration polarization or high current density region.

## Figures and Tables

**Table 1:** Boundary conditions in the PSM.

Parameters	$\mathrm{Side}\;\mathrm{Near}\;\mathrm{GDL}\;b_{1}$	$\mathrm{Side}\;\mathrm{Near}\;\mathrm{CL}\;b_{2}$
$c_{O_{2}}/$mol m^−3^	8	7.9
$c_{H_{2}O}$/mol m^−3^	9.9	10
$\phi_{s}/$V	0.75	0.76
T/K	355.9	356

**Table 2:** Correlation of MPL transport properties with compression strain and porosity.

	In-Plane				Through-Plane			
	Equation (Porosity)	R^2^	Equation (Strain)	R^2^	Equation (Porosity)	R^2^	Equation (Strain)	R^2^
$\frac{D}{D_{eff}}$	$0.562\varepsilon^{-3.16}$	97.5%	$3.5e^{6.45\tau }$	98.8%	$0.62\varepsilon^{-3.01}$	96.8%	$3.64e^{6.39\tau }$	98.4%
τ	$0.562\varepsilon^{-2.16}$	96.5%	$2.05e^{3.99\tau }$	98.6%	$0.62\varepsilon^{-2.07}$	95.4%	$2.13e^{3.86\tau }$	98.2%
*k* (W m^−1^ K^−1^)	$1.06e^{-2.7\varepsilon }$	99.8%	$0.25e^{1.95\tau }$	99.5%	$1.06e^{-2.68\varepsilon }$	99.7%	$0.26e^{1.93\tau }$	99.3%
σ (S m^−1^)	$15,172e^{-5.51\varepsilon }$	98.97%	$810e^{3.99\tau }$	99.8%	$14,678e^{-5.4\varepsilon }$	99.2%	$831e^{3.9\tau }$	99.8%
κ (um^2^)	$6\;\times \;10^{-4}e^{14.49\varepsilon }$	99.8%	$1.18e^{-10.3\tau }$	97.3%	$4\;\times \;10^{-4}e^{15.32\varepsilon }$	97.5%	$1.185e^{-10.9\tau }$	97.8%
*S* (kPa)	$0.48e^{-1.44\times 10^{-5}Pc}$ $\;-\;0.47e^{-5.1\times 10^{-4}Pc}$			98.3%	$0.48e^{-1.46\times 10^{-5}Pc}$ $\;-\;0.47e^{-5\times 10^{-4}Pc}$			99.1%

## Data Availability

Not applicable.
